# Esthetic Rehabilitation of Primary Anterior Teeth using Temporization Material: A Novel Approach

**DOI:** 10.5005/jp-journals-10005-1418

**Published:** 2017-02-27

**Authors:** Neeraj Gugnani, IK Pandit, Monika Gupta, Jyoti Nagpal

**Affiliations:** 1 Professor, Department of Pedodontics and PCD, JN Kapoor D.A.V Centenary Dental College, Yamuna Nagar, Haryana, India; 2 Professor and Head, Department of Pedodontics and PCD, JN Kapoor D.A.V Centenary Dental College, Yamuna Nagar, Haryana, India; 3 Professor, Department of Pedodontics and PCD, JN Kapoor D.A.V Centenary Dental College, Yamuna Nagar, Haryana, India; 4 Postgraduate Student, Department of Pedodontics and PCD, JN Kapoor D.A.V Centenary Dental College, Yamuna Nagar, Haryana, India

**Keywords:** Early childhood caries, Esthetic rehabilitation, Primary tooth trauma, Strip crown, Temporization material.

## Abstract

Pediatric dentists should always aim for esthetic and functional rehabilitation of decayed/traumatized primary teeth. The most common method for restoring such teeth involves the use of “strip crowns” with composites, while the recent trend is toward using other extracoronal restorations including preve-neered stainless steel crowns and zirconia crowns. All these restorative options have shown good success rates, but also have some limitations. This case series depicts novel clinical technique of using a temporization material for full-coronal restoration(s) in primary anterior teeth. This included the chair-side custom fabrication of full-coronal restoration using temporization material, which has resulted in good immediate esthetics and might be a cost-effective alternative for restoring primary anterior teeth in future.

**How to cite this article:**

Gugnani N, Pandit IK, Gupta M, Nagpal J. Esthetic Rehabilitation of Primary Anterior Teeth using Temporization Material: A Novel Approach. Int J Clin Pediatr Dent 2017;10(1):111-114.

## INTRODUCTION

The most common problems in childhood leading to structural damage of primary maxillary anterior teeth include early childhood caries and dental trauma. Structural loss of these teeth not only affects esthetics, but also leads to compromised mastication, poor phonetics along with a difficulty in social and physiological adjustment.^1^ Hence, esthetic and functional rehabilitation of these decayed/traumatized primary teeth should always be the main treatment objective. Such teeth can be restored using either intracoronal/full-coronal restorations. Intra-coronal restorations are indicated for single-surface caries and include tooth-colored materials like composites, glass ionomer cement, etc,^2^ while for situations like teeth with multisurface caries involvement and traumatized/ discolored teeth, full-coronal restorations are indicated.^3^

The most common method for restoring such teeth involves the use of “strip crowns” with composites.^4^ These restorations exhibit good esthetics and high success rate,^4^ but difficult moisture control, decreased surface area, and technique sensitivity frequently compromise the retention and success of these restorations.^5^ Other full-coronal restorations include prefabricated zirconia, preveneered stainless steel, and polycarbonate crowns.^3^ These crowns exhibit better esthetics and retention, but higher cost and difficult adaptation are some of their limitations.^3^ Hence, an alternative would be to fabricate chair-side, customized full-coronal restoration, which should also be esthetic, durable, and cheap. This case series depicts novel application of temporization material for full-coronal restoration(s) in primary anterior teeth.

## MATERIALS AND METHODS

In recent times, bis-acryl composite-based temporization material has become the material of choice for temporiza-tion purposes owing to its improved mechanical properties. One such bis-acryl composite temporary material is available as Luxatemp Star (DMG, Germany). It is available as two-component material, which are mixed just prior to its use, using the automix syringe that dispenses the material in the ratio of 10:1. It is available in different shades, and thus helps to achieve customized esthetics suiting the patient’s needs. Though it is frequently used for temporization of permanent teeth, it has also been reported as a treatment option for restoring primary teeth.^6^ In this case series, patients visiting the outpatient department of Pedodontics at JN Kapoor D.A.V Centenary Dental College, Yamuna Nagar, Haryana, India, were screened, and those requiring esthetic rehabilitation of primary anterior teeth, owing to structural loss/discoloration due to caries or trauma, were selected. Esthetic rehabilitation of the patient was planned using the custom-fabricated crowns with temporization material. Approval for the procedure was sought from the Institutional Review Board of JN Kapoor D.A.V Centenary Dental College Yamuna Nagar, Haryana, India. Informed consent was sought from the parents after explaining to them about the treatment procedure and available alternatives.

**Figs 1A to F:**
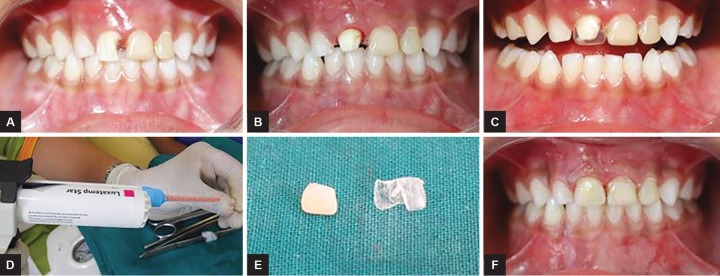
(A) Carious broken primary anterior tooth # 51; (B) caries excavation followed by blocking of undercuts and tooth preparation; (C) selection of strip crown; (D) loading the strip crown with temporization material; (E) strip crown teared off, crown prepared; (F) cemented crown, immediate postoperative esthetics

The first case was a 4-year-old male patient who reported with a chief complaint of carious 51 (Fig. 1A). Following steps were followed for this novel approach of restoring the tooth with custom-fabricated crowns with temporization material:

- *Step 1: Caries excavation and composite core build-up (if required):* Initially, caries was excavated and composite core-build-up was done, to block the undercuts.
- *Step 2: Tooth preparation:* Crown cutting was done, reducing the tooth ~1.5 mm from all sides (Fig. 1B).
- *Step 3: Selection of appropriate-sized strip crowns:* An appropriate-sized strip crown was selected to fabricate the crown. This is done similar to the strip-composite technique (Fig. 1C).
- *Step 4: Strip crown loading:* Shade matching was done in accordance with the adjacent teeth, and strip crown was loaded with temporization material using automix syringe. Loaded strip crowns were then placed on the prepared tooth within 45 seconds and removed while the material is still in elastic stage (1.30-2.20 minutes after start of mixing) (Fig. 1D).
- *Step 5: Tearing of the strip crown:* Material was then allowed to set extraorally, and strip crown was peeled off (Fig. 1E).
- *Step 6: Crown cementation:* The finished crown was cemented over the prepared crown using Perma-cem^TM^ (DMG, Germany). Good immediate esthetic results were achieved along with the good satisfaction among parents (Fig. 1F).

**Figs 2A to D:**
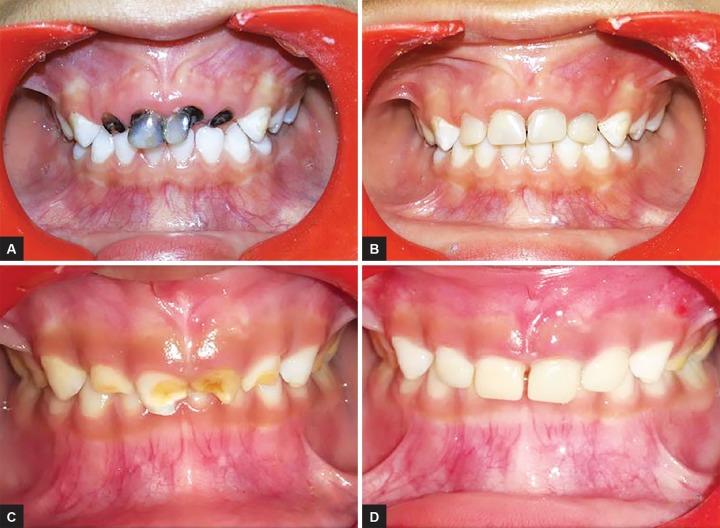
Esthetic rehabilitation of two cases of carious broken anterior teeth

**Figs 3A to D:**
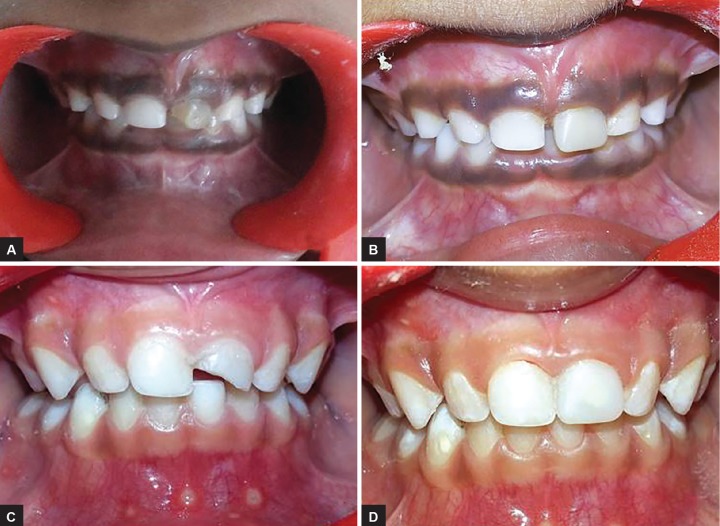
Esthetic rehabilitation of two cases of broken and traumatized primary anterior teeth

Esthetic rehabilitation of other patients with caries and trauma was also done using the similar custom-fabricated crowns using temporization material (Luxatemp Star). In cases the teeth required endodontic treatment, it was completed before the fabrication of crowns. Figures 2A to D and 3A to D show the esthetic rehabilitation of two cases of carious broken and traumatized primary anterior teeth respectively.

## DISCUSSION

The crowns used for restoring primary incisors can be broadly categorized as those that get bonded to the tooth and those that are cemented using luting cement.^3^ Bonded strip crowns have been the choice of dentists worldwide owing to excellent esthetics and ease of repair;^7^ on the contrary, these restorations may discolor, break, or fail due to lack of appropriate bonding, owing to less surface area.^4,8^

Alternatively, among the cemented-crown category, preveneered SS and zirconia crowns are being frequently advocated owing to their superior esthetics and dura-bility.^3,9^ However, these crowns require excessive tooth cutting, are difficult to adapt due to inability to crimp, and are very costly.^3,10^

The crowns fabricated in this case series have used a temporization material. The rationale behind using temporization material for crown fabrication in primary teeth is that primary teeth need to be retained only for a limited period of time, so that a material, i.e., durable enough for “this period” may prove to be a suitable alternative. Moreover, in contrast to using strip crown with composite, a crown fabricated with temporization material is not dependent on the direct bonding of composite to the tooth material and thus, may have better retention properties even in cases where remaining tooth structure is less. This bis-acrylate-based temporization material (Luxatemp Star) is available in different shades and claims to have good flexural strength, better color stability, and dimensional stability. All these properties make it a suitable choice for esthetic restoration of primary carious teeth. Custom fabrication further ensured good adaption at the margins, and the final restorations exhibited good immediate esthetics.

Conclusively, chair-side custom fabrication of full-coronal restoration in primary anterior teeth using a temporization material might be a cost-effective alternative; however, randomized clinical trials are required to evaluate color stability and longevity of these restorations.

## CLINICAL SIGNIFICANCE

- Pediatric dentists have to frequently use full-coronal restorations for esthetic rehabilitation of primary anterior teeth using either of the available options, e.g., strip crown technique, zirconia crowns, preveneered SS crowns, etc.
- Custom-fabricated crowns using temporization material seem to be an esthetic and cost-effective alternative for restoring primary anterior teeth.

## References

[1] Davies GN (1998). Early childhood caries - a synopsis.. Community Dent Oral Epidemiol.

[2] Walia T, Salami AA, Bashiri R, Hamoodi OM, Rashid F (2014). A randomised controlled trial of three aesthetic full-coronal restorations in primary maxillary teeth.. Eur J Paediatr Dent.

[3] Waggoner WF (2015). Restoring primary anterior teeth: updated for 2014.. Pediatr Dent.

[4] Ram D, Fuks AB (2006). Clinical performance of resin-bonded composite strip crowns in primary incisors: a retrospective study.. Int J Paediatr Dent.

[5] Waggoner WF (2002). Restoring primary anterior teeth.. Pediatr Dent.

[6] Burykina M, Admakin O, Skatova E, Medvedeva T, Kozlova N (2013). Indirect restoration of primary teeth with individual crowns.. Int J Paediatr Dent.

[7] Waggoner WF (2006). Anterior crowns for primary anterior teeth: an evidence based assessment of the literature.. Eur Arch Paediatr Dent.

[8] Duhan H, Pandit IK, Srivastava N, Gugnani N, Gupta M, Kochhar GK (2015). Clinical comparison of various esthetic restorative options for coronal build-up of primary anterior teeth.. Dent Res J (Isfahan).

[9] Dhar V, Hsu KL, Coll JA, Ginsberg E, Ball BM, Chhibber S, Johnson M, Kim M, Modaresi N, Tinanoff N (2015). Evidence-based update of pediatric dental restorative procedures: dental materials.. J Clin Pediatr Dent.

[10] Soxman JA. (2015). Handbook of clinical techniques in pediatric dentistry..

